# Supplementary material for: Nine years of in situ soil warming and topography impact the temperature sensitivity and basal respiration rate of the forest floor in a Canadian boreal forest
Source: PLoS One. 2019 Dec 26;14(12):e0226909. doi: 10.1371/journal.pone.0226909 (PMC6932772; doi:10.1371/journal.pone.0226909)
Supplement: S1 Table — Q10 and B parameters values are shown for each landform unit of the four experimental plots (C, N+, W+ and W+N+) and in outside-plot samples after nine years of treatment. Mean values (± SD) for each treatment are shown in bold. (DOCX) [file pone.0226909.s001.docx]

**S1 Table. Q_10_ and B values in the forest floor of each plot**

Q_10_ and B parameters values are shown for each landform unit of the four experimental plots (C, N+, W+ and W+N+) after nine years of treatment. Parameters values are shown for models fitted with (40+) and without (40-) the respiration rate (RR) values obtained from the incubation at 40°C. Mean values (± SD) for each treatment are shown in bold.

| Treatment | Slope position | Q_10_ | | B | |
| --- | --- | --- | --- | --- | --- |
|  |  | 40+ | 40- | 40+ | 40- |
| C | Upper | 2.29 | 2.01 | 2.79 | 3.56 |
| C | Back | 3.22 | 2.46 | 1.50 | 3.51 |
| C | Lower | 1.84 | 3.00 | 8.99 | 2.09 |
| C |  | **2.45 ± 0.70** | **2.49 ± 0.50** | **4.43 ± 4.0** | **3.05 ± 0.83** |
| N+ | Upper | 2.16 | 3.00 | 3.84 | 1.62 |
| N+ | Back | 1.80 | 3.32 | 7.62 | 1.48 |
| N+ | Lower | 1.99 | 1.82 | 4.89 | 5.89 |
| N+ |  | **1.98 ± 0.18** | **2.71 ± 0.79** | **5.45 ± 1.96** | **3.00 ± 2.50** |
| W+ | Upper | 3.97 | 2.46 | 0.68 | 2.67 |
| W+ | Back | 3.63 | 2.46 | 0.78 | 2.64 |
| W+ | Lower | 3.00 | 3.67 | 2.23 | 1.38 |
| W+ |  | **3.54 ± 0.48** | **2.86 ± 0.70** | **1.23 ± 0.90** | **2.23 ± 0.74** |
| W+N+ | Upper | 4.13 | 2.72 | 0.67 | 2.35 |
| W+N+ | Back | 2.48 | 3.00 | 3.43 | 1.99 |
| W+N+ | Lower | 2.43 | 4.05 | 3.44 | 0.87 |
| W+N+ |  | **3.01 ± 0.96** | **3.26 ± 0.70** | **2.51 ± 1.60** | **1.74 ± 0.77** |
| Warmed | NA | **3.28 ± 0.74** | **3.06 ± 0.66** | **1.87 ± 1.35** | **1.98 ± 0.73** |
| Unwarmed | NA | **2.24 ± 0.56** | **2.60 ± 0.60** | **4.94 ± 2.87** | **3.03 ± 1.67** |
| Fertilized | NA | **2.51 ± 0.80** | **2.99 ± 0.73** | **3.98 ± 2.26** | **2.37 ± 1.80** |
| Unfertilized | NA | **3.02 ± 0.85** | **2.68 ± 0.58** | **2.83 ± 3.13** | **2.64 ± 0.84** |
