# Supplementary material for: Nine years of in situ soil warming and topography impact the temperature sensitivity and basal respiration rate of the forest floor in a Canadian boreal forest
Source: PLoS One. 2019 Dec 26;14(12):e0226909. doi: 10.1371/journal.pone.0226909 (PMC6932772; doi:10.1371/journal.pone.0226909)
Supplement: S2 Table — Q10 and B parameters values for each landform unit outside the experimental plots after nine years of treatment. Mean values (± SD) for each treatment are shown in bold. (DOCX) [file pone.0226909.s002.docx]

**S2 Table. Q_10_ and B values in the forest floor of outside plot**s

Q_10_ and B parameters values for each landform unit outside the experimental plots after nine years of treatment. Mean values (± SD) for each treatment are shown in bold.

| Treatment | Slope position | Q_10_ | B |
| --- | --- | --- | --- |
| Outside | Upper | 3.60 | 0.73 |
| Outside | Back | 1.99 | 4.75 |
| Outside | Lower | 1.86 | 8.36 |
| Outside | Upper | 2.03 | 6.74 |
| Outside | Back | 1.68 | 8.30 |
| Outside | Lower | 1.82 | 7.99 |
| Outside | Upper | 1.91 | 4.51 |
| Outside | Back | 2.91 | 2.36 |
| Outside | Lower | 2.12 | 5.69 |
| Outside | Upper | 1.99 | 4.98 |
| Outside | Back | 1.93 | 6.27 |
| Outside | Lower | 1.72 | 8.74 |
|  |  | **2.13 ± 0.56** | **5.78 ± 2.49** |
