# Supplementary material for: Nine years of in situ soil warming and topography impact the temperature sensitivity and basal respiration rate of the forest floor in a Canadian boreal forest
Source: PLoS One. 2019 Dec 26;14(12):e0226909. doi: 10.1371/journal.pone.0226909 (PMC6932772; doi:10.1371/journal.pone.0226909)
Supplement: S3 Table — Values (mean ± SD; n = 3) are shown for samples collected from the four treatments (C, N+, W+ and W+N+) after nine years of the in-situ experiment. No significant differences were found between treatments for any of the studied variables (one-way ANOVA; P > 0.05). Organic matter (OM) content was measured by weight loss on ignition (360 °C), total N and C contents by combustion, and P, K, Ca, Mg, Mn, Cu, Zn, Al, Fe and S concentrations by ICP-AES following Mehlich 3 extraction method. (DOCX) [file pone.0226909.s003.docx]

**S3 Table. Forest floor chemical composition and characteristics.**

Values (mean ± SD; n=3) are shown for samples collected from the four treatments (C, N+, W+ and W+N+) after nine years of the in-situ experiment. No significant differences were found between treatments for any of the studied variables (one-way ANOVA; *P* > 0.05). Organic matter (OM) content was measured by weight loss on ignition (360 °C), total N and C contents by combustion, and P, K, Ca, Mg, Mn, Cu, Zn, Al, Fe and S concentrations by ICP-AES following Mehlich 3 extraction method.

|  | C | N+ | W+ | W+N+ |
| --- | --- | --- | --- | --- |
| OM (g kg^-1^) | 826.3 ± 112.1 | 934 ± 12.1 | 911.3 ± 57.5 | 879.3 ± 50.9 |
| Moisture (%) | 9.3 ± 1.2 | 10.1 ± 0.1 | 10 ± 0.8 | 9.7 ± 0.6 |
| C (g kg^-1^) | 430 ± 54.5 | 490 ± 25.2 | 475.7 ± 27.5 | 467 ± 29.8 |
| N (g kg^-1^) | 12.2 ± 1.8 | 11.9 ± 0.9 | 12.6 ± 1.1 | 12 ± 0.2 |
| pH (H_2_O) | 4.0 ± 0.2 | 3.8 ± 0.1 | 3.8 ± 0.1 | 3.8 ± 0.1 |
| P (mg kg^-1^) | 145.3 ± 45.1 | 150.7 ± 17 | 151 ± 21.8 | 140.7 ± 38.7 |
| K (mg kg^-1^) | 492.3 ± 149.1 | 527.3 ± 126.4 | 520.3 ± 80.6 | 474 ± 68.5 |
| Ca (mg kg^-1^) | 1477.7 ± 632.7 | 1593.3 ± 509.6 | 1670 ± 91.7 | 1273.3 ± 363 |
| Mg (mg kg^-1^) | 435 ± 216.8 | 334.3 ± 40.1 | 384.7 ± 114 | 301 ± 45.7 |
| Mn (mg kg^-1^) | 48 ± 20.3 | 60 ± 33 | 59.3 ± 9.3 | 36.3 ± 13.1 |
| Cu (mg kg^-1^) | 40.7 ± 33.3 | 19.7 ± 15.9 | 35.7 ± 22.6 | 27.3 ± 12 |
| Zn (mg kg^-1^) | 144.7 ± 39.7 | 117.7 ± 46.3 | 132.7 ± 49.7 | 113.7 ± 45.6 |
| Al (mg kg^-1^) | 940 ± 198 | 789.3 ± 797.3 | 770.7 ± 493.4 | 1181.7 ± 798.6 |
| Fe (mg kg^-1^) | 434.3 ± 300.7 | 342.3 ± 330.8 | 293.3 ± 193.5 | 398.7 ± 357.2 |
| S (mg kg^-1^) | 62.7 ± 6.7 | 65 ±13.5 | 64 ± 5.2 | 63.7 ± 13.5 |
