# Supplementary material for: Nine years of in situ soil warming and topography impact the temperature sensitivity and basal respiration rate of the forest floor in a Canadian boreal forest
Source: PLoS One. 2019 Dec 26;14(12):e0226909. doi: 10.1371/journal.pone.0226909 (PMC6932772; doi:10.1371/journal.pone.0226909)

**S1 Figure. Differences in soil temperature (Δ Temperature) between unwarmed and warmed plots.**

Differences on a daily basis in (a) the upper slope and (c) the back slope. Differences on a monthly basis (mean ± SD) in (b) the upper slope and (d) the back slope between 2008 and 2018. Months 1 and 12 are January and December, respectively.


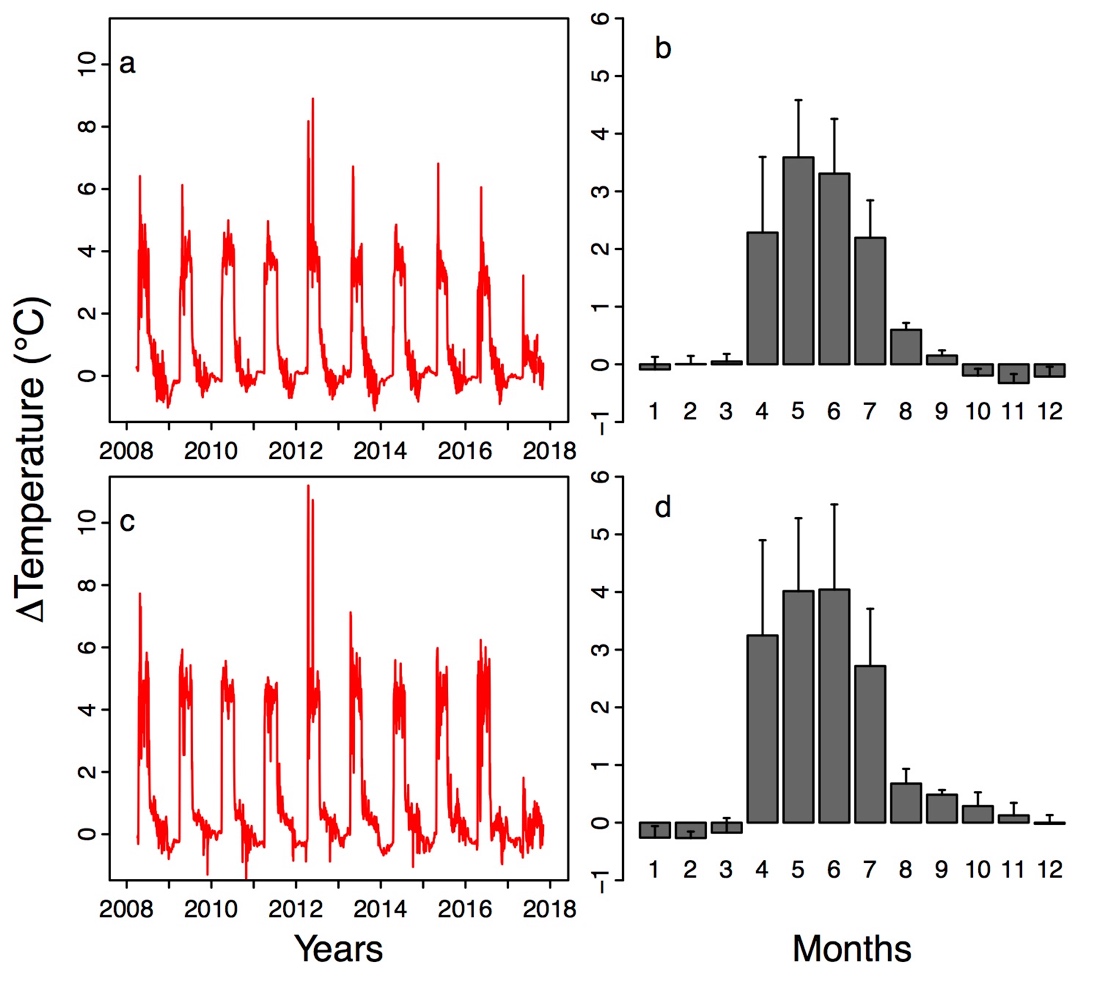

Supplement: S1 Fig — Differences on a daily basis in (a) the upper slope and (c) the back slope. Differences on a monthly basis (mean ± SD) in (b) the upper slope and (d) the back slope between 2008 and 2018. Months 1 and 12 are January and December, respectively. (DOCX) [file pone.0226909.s004.docx]
