# Supplementary material for: Nine years of in situ soil warming and topography impact the temperature sensitivity and basal respiration rate of the forest floor in a Canadian boreal forest
Source: PLoS One. 2019 Dec 26;14(12):e0226909. doi: 10.1371/journal.pone.0226909 (PMC6932772; doi:10.1371/journal.pone.0226909)

**S2 Figure. Relationship between forest floor mean respiration rate (Forest floor RR, µg C-CO_2_·g^-1^·C·h^-1^) and incubation temperatures (°C) for soil samples collected outside the experimental plots.**

Relationships are shown for the four experimental treatments (C: control; N+: CNA; W+: soil warming; W+N+: combined soil warming and CNA). The rows and the columns show the treatments and replicates for each treatment, respectively. Curves were obtained by fitting a first-order exponential equation (RR = Be^k.T^).


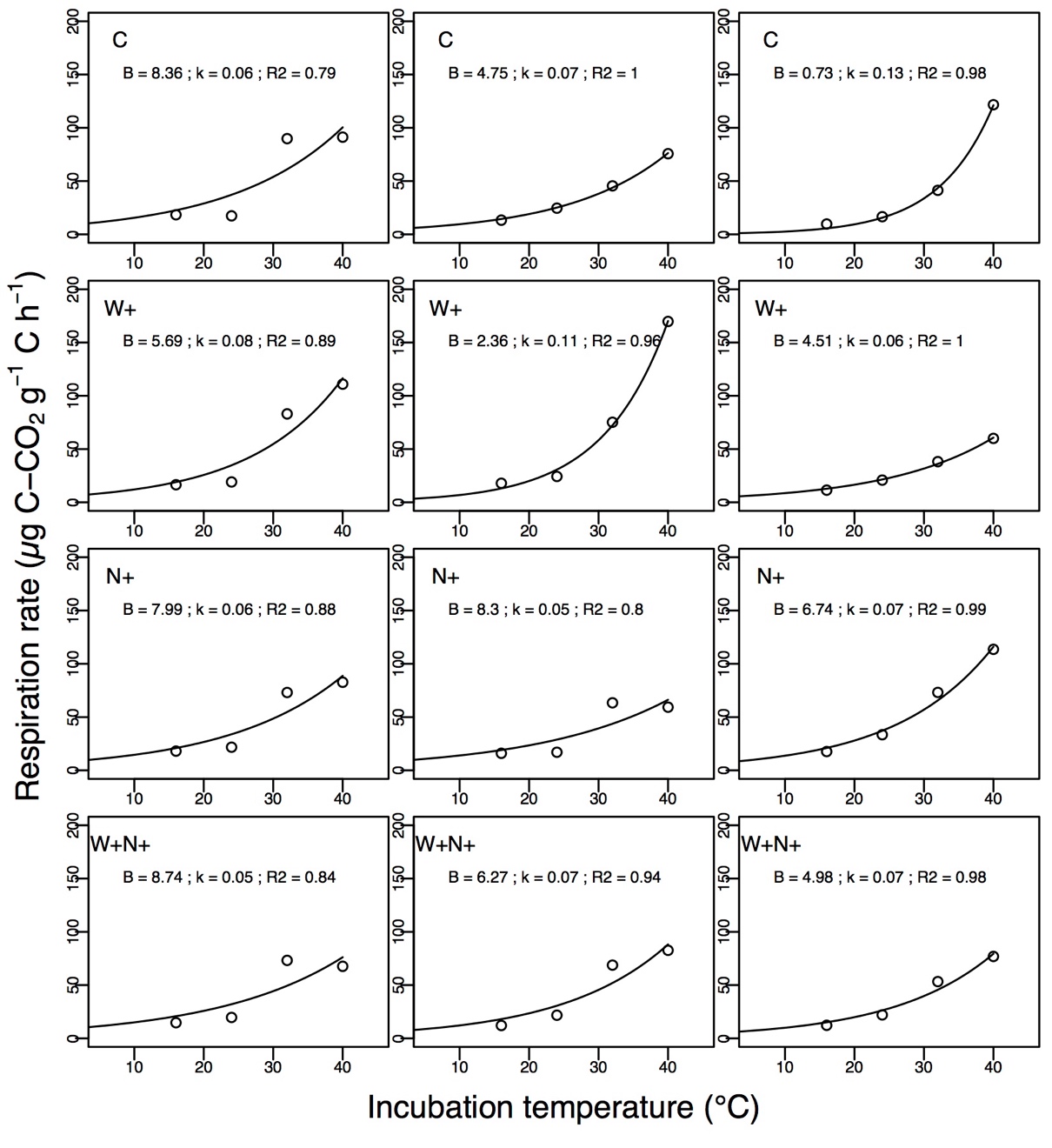

Supplement: S2 Fig — Relationships are shown for the four experimental treatments (C: control; N+: CNA; W+: soil warming; W+N+: combined soil warming and CNA). The rows and the columns show the treatments and replicates for each treatment, respectively. Curves were obtained by fitting a first-order exponential equation (RR = Bek.T). (DOCX) [file pone.0226909.s005.docx]
