# Supplementary material for: Nine years of in situ soil warming and topography impact the temperature sensitivity and basal respiration rate of the forest floor in a Canadian boreal forest
Source: PLoS One. 2019 Dec 26;14(12):e0226909. doi: 10.1371/journal.pone.0226909 (PMC6932772; doi:10.1371/journal.pone.0226909)
Supplement: Attachment — Submitted filename: Response to the reviewers.docx [file pone.0226909.s008.docx]

Please include the following items when submitting your revised manuscript:

- A rebuttal letter that responds to each point raised by the academic editor and reviewer(s). This letter should be uploaded as separate file and labeled 'Response to Reviewers'.
- A marked-up copy of your manuscript that highlights changes made to the original version. This file should be uploaded as separate file and labeled 'Revised Manuscript with Track Changes'.
- An unmarked version of your revised paper without tracked changes. This file should be uploaded as separate file and labeled 'Manuscript'.

Please note while forming your response, if your article is accepted, you may have the opportunity to make the peer review history publicly available. The record will include editor decision letters (with reviews) and your responses to reviewer comments. If eligible, we will contact you to opt in or out.

We look forward to receiving your revised manuscript.

Kind regards,

Julian Aherne
Academic Editor
PLOS ONE

6. Review Comments to the Author

Please use the space provided to explain your answers to the questions above. You may also include additional comments for the author, including concerns about dual publication, research ethics, or publication ethics. (Please upload your review as an attachment if it exceeds 20,000 characters)

**Reviewer #1:**

**The manuscript has seen a rigorous review and the comments of the reviewers have been addressed with authority and patience. The text has the potential to be widely cited.**

**Reviewer #2:**

**All comments made by previous reviewers have been addressed adequately in the rebuttal. However, the abstract is confusing and seems contradictory in places. Please ensure that the language used there is consistent with that of the rest of the manuscript. Specifically, the observation that there is no effect of treatments on soil chemistry is brought up in both lines 37-39 and 45-47. Remove one.** **On L49, it appears the authors have confused chemical quality/recalcitrance again, saying that in contrast slope position has an effect on FF organic C quality. But "Organic C quality" is also used on line 42 to talk about the B parameter (as distinct from NMR measurements of quality), and on line 53-54 the authors say topography and soil warming both affect soil C quality. It seems like the topology position overwhelmed the warming effect, so maybe this should be emphasized**.

Response:

We have significantly modified the abstract in accordance with reviewer #3 comments. We have removed the term “C recalcitrance” from the text in most places and replaced it by C quality.

**Reviewer #3:**

**The revised manuscript has improved, but there are still some issues. The abstract is long-winded and needs to be shortened and cleared (for an example see below). The text is more self-critical now with regard of findings with and without the 40°C step, but the most interesting info is missing -> Is the warming effect on Q10 and B still statistically significant if the 40°C step is not considered? This needs to be clearly stated!**

Response: The same trend is observed when the incubation at 40°C is removed but the difference is not statistically significant anymore, probably because of the small number of replicates and the large variability among replicates. We have dedicated a full paragraph to this issue in the discussion:

L. 682: “Although our data show a clear impact of soil warming on both B and the Q_10_ of forest floor RR, our results may have been slightly different with another range of incubation temperatures. Boreal forest floors never experience temperatures as high as 40°C. These conditions may have somehow perturbed soil microorganisms and modified their metabolic activity. Therefore, parameter values may have been slightly different if the samples had been incubated at lower temperatures. As shown in Fig. 2, B and k parameters were sometimes significantly different when the incubations at 40°C were not included in the analysis. Although the trends were similar (i.e., higher Q_10_ and lower B in warmed plots as compared to unwarmed plots), the effect of soil warming on Q_10_ and B was not significant after the values of the incubation at 40°C were removed from the data set (S1 Table).”

**There are some typos in the new text (see below). More care needs to be taken when taking about temperature sensitivity (e.g. heading L447-448, heading L248-249 and elsewhere). Only the temperature sensitivity of RR was calculated – not the temperature sensitivity of forest floor organic C or anything else. Please reword and correct this. I am not sure if the ultimate conclusion is justified by the data.**

Response:

We agree with the reviewer. We have changed it throughout the text.

**How can RR increase in future when 9 years of strong warming had no effects on forest floor C contents or quality and RR generally is lower at warmed plots (lower B means also lower respiration at low temperatures – and in boreal forests most of the year is low temperatures)? The higher temperature sensitivity seems to play a comparable little role…**

Response:

We agree that it is speculative. This is however a theoretically possible given our results. Nine years of soil warming have resulted in an increase in the temperature sensitivity of forest floor RR, without perceptible change in C composition or recalcitrance per se. Ten additional years of warmer temperatures due to climate change may accentuate this trend and further increase RR temperature sensitivity. Higher CO2 efflux from the soil may therefore occur during hot summer days, which integrated over a long period of time, may eventually impact soil organic C content.

We also agree that cold temperatures prevail most of the year in boreal forests. However, CO2 fluxes during the cold period are probably negligible because the rates of biochemical processes are very slow. Our incubations show that the RR is several times higher at 24°C than at 0°C. In addition, climate models predict an extension of the growing season duration.

We however have agreed to remove this statement, which is probably too speculative.

**… Still the reader has no idea if RR is measured in the field and if any field response to warming was observed.**

We have added a sentence in the M&M section and one in the discussion:

(L. 243): “No RR measurements were conducted in the field. All measurements were performed in the laboratory after forest floor samples were incubated at different temperatures.”

(L. 528): “We cannot confirm this hypothesis from the present data as no RR were measured in situ.”

**The new title is fine.**

**Here comes an example for an comprehensive abstract – you may use it or parts of it if you agree. Not all details must be written there – for methodological details, the reader can look up the text…

Example:

The forest floor of boreal forest stores large amounts of organic C that may react to a warming climate and increased N deposition. It is therefore crucial to assess the impact of these factors on the temperature sensitivity of this C pool to help predict future soil CO2 emissions from boreal forest soils to the atmosphere. In this study, in-situ soil warming (+2–4 °C) and canopy N addition (CNA; +0.30–0.35 kg•N•ha-1•yr-1) plots were replicated along a topographic gradient (upper, back and lower slope) in a boreal forest in Quebec, Canada. After nine years of treatment, forest floor was collected from each plot, and its organic C quality was characterized through solid-state 13C nuclear magnetic resonance (NMR) spectroscopy. Forest floor samples were incubated at 16, 24, 32 and 40°C and respiration rates (RR) were measured to assess the temperature sensitivity (Q10) and basal respiration rates (B) of RR. Both, soil warming and CNA had no significant effect on forest floor chemistry (e.g., C,N, Ca and Mg content, amount of soil organic matter, pH, chemical functional groups). The NMR analyses did not show evidence of significant changes in the forest floor organic C quality. Nonetheless, a significant effect of soil warming on both the Q10 and B was observed. On average, B was 72% lower and Q10 45% higher in the warmed, versus the control plots. CNA had no significant effect on the measured soil and respiration parameters and no interaction effects with warming. In contrast, slope position had a significant effect on forest floor organic C quality. Upper slope plots had higher soil alkyl C:O-alkyl C ratios and lower B values than those in the lower slope, across all different treatments. Our results point towards higher temperature sensitivity of RR under warmer conditions, accompanied by an overall down-regulation of RR at low temperatures (lower B). Since soil C quantity and quality were unaffected by the 9 years warming, the observed patterns could result from microbial adaptations to warming.**

**If you agree with the logic of that, the discussion and conclusion needed to be adopted a bit accordingly. If you disagree, you might at least take over the shortened passages at the beginning…**

Response:

Thank you very much for this. It’s excellent. We have replaced the previous abstract by this one and added a few sentences from the previous one.

**Intro is fine.

Materials and Methods – L222-229 belong to the discussion.**

Response:

Ok. We have moved this paragraph to the first section of the discussion (L. 461). We also added a paragraph here: “As shown on Fig 2, the removal of the incubation at 40°C had a sometimes a significant effect on the rate of increase of RR with incubation temperature and the RR at 0°C (k and B parameters, respectively), which suggests that the chosen range of incubation temperature can impact the results in this type of study.” (L. 457)

**Discussion: (the line numbers belong to the version with corrections at the end of the pdf)
L470: delete “tends”**

Ok

**L472: ..decrease in B caused by…**

Ok

**L477: it might not solely be a matter of changes in microbial composition. Microbial physiology can as well change if composition does not shift. You may add a reference on proteomics here (62-64) e.g. Liu, Dong, et al. "Microbial functionality as affected by experimental warming of a temperate mountain forest soil—a metaproteomics survey." Applied soil ecology 117 (2017): 196-202. And change the text to ….shifts in soil microbial decomposition and/or physiology, which impact….**

Response: We have added the reference and the following sentence:

(L. 537). “The decrease in B may instead have resulted from changes in abiotic factors such as substrate or nutrient availability, as well as from changes in soil microbial composition and activity. Soil warming can indeed induce shifts in soil microbial populations and species [61–63], as well as in microbial physiological functioning [64], which both impact the respiration rate-temperature relationship and C substrate use efficiency. Therefore, the possibility of a microbial shift, which reduced respiration rate at low temperature over the nine years of the experiment could be an explanation of the observed decrease in B in warmed plots.”

**L481: … the nine years of the experiment could be an explanation of the observed decrease in B.**

Ok.

**L573: issue with temperature sensitivity wording – see above**

Response:

We have changed it: “Effect of N addition on the temperature sensitivity of forest floor’s respiration rate and organic C chemistry”

**Conclusions might be adapted as mentioned above.**

Response:

The conclusion has been modified according to reviewer’s previous comments.

**Table S1 and S2 – why still “recalcitrancy”? in the legend**

Response:

Sorry for this error. We have changed the captions. “Temperature sensitivity (Q_10_) and basal rate (B) of forest floor respiration”.

**For the figures main figures, I could not find any captions in the pdf**

Response:

In Plosone, the captions are included within the text rather than at the end of the manuscript.

**Reviewer #4: The authors have answered all the questions. Thanks for making necessary changes. I recommend publication in PLOS ONE.**
